# Electrospinning of Carboxymethyl Chitosan/Polyoxyethylene Oxide Nanofibers for Fruit Fresh-Keeping

**DOI:** 10.1186/s11671-018-2642-y

**Published:** 2018-08-15

**Authors:** Tian-Tian Yue, Xiao Li, Xiao-Xiong Wang, Xu Yan, Miao Yu, Jian-Wei Ma, Yu Zhou, Seeram Ramakrishna, Yun-Ze Long

**Affiliations:** 1 Collaborative Innovation Center for Nanomaterials & Devices, College of Physics, Qingdao University, Qingdao, 266071 China; 2 Industrial Research Institute of Nonwovens & Technical Textiles, College of Textiles & Clothing, Qingdao University, Qingdao, 266071 China; 3 Department of Mechanical Engineering, Columbia University, New York, NY 10027 USA; 4 Medical College, Qingdao University, Qingdao, 266071 China; 5 Center for Nanofibers & Nanotechnology, Nanoscience & Nanotechnology Initiative, Faculty of Engineering, National University of Singapore, Singapore, Singapore

**Keywords:** Electrospinning, Fresh-keeping, Strawberries, Nanofiber membrane

## Abstract

Electrospinning provides an effective method for generating nanofibers from solution of carboxymethyl chitosan/polyoxyethylene oxide (CMCS/PEO). The goal of this work is to explore the potential application of electrospun CMCS/PEO nanofiber membrane in fruit fresh-keeping. The microstructure, antibacterial activity, hydrophilia, and air permeability of the nanofiber membrane have been tested. For comparison, the fresh-keeping effects of commercial cling wrap and CMCS/PEO nanofiber membranes on strawberries’ rotting rate and weight loss rate have been studied. The results indicate that the electrospun CMCS/PEO membrane could effectively avoid water loss in strawberries and has a remarkable effect to prolong strawberries’ shelf life due to its breathability and antibacterial activity. In addition, the composite CMCS/PEO, nanofiber membrane is non-poisonous and edible, which can be used in food industry.

## Background

The development of food protecting films and coatings were turned from physical or mechanical treatments to chemical protection. People focus on biological materials which most possess edibility such as proteins, lipids, and polysaccharide instead of traditional protective film like plastic, papers and paraffin [1, 2]. With the growing awareness of environmental protection, edible coatings and films can be extensively used in food especially for fruits and vegetables which need highly effective maintained freshness. Simple coating even a thin layer of membrane with some specific features may achieve better effects [3]. Chitosan is an excellent natural fit in food due to its biodegradability, biocompatibility, antimicrobial activity, non-toxicity, versatile chemical, and physical properties [4, 5], and with its unique antibacterial property, rot resistance, and film-forming property, it has been widely used in medicine, textile and food [6–9]. Particularly, chitosan can be derived from the raw materials of silkworm, shrimp, and crab shells, which are widely and abundantly distributed in nature [10].

Electrospinning, which continuously fabricates soft nanofibrous membranes [11, 12], can offer gentle protection to fruits. This may help to solve the storage and transportation problems of some fruits such as strawberry, cherry tomatoes, and kumquat. With a layer of soft nanofibers, the surface of the fruit can be protected from the outside invasion, like the introduction of bacteria and scratches. In several studies, concentrated acetic acid solution was used as a solvent for electrospinning chitosan nanofibers, and carboxymethyl chitosan (CMCS) electrospun nanofibers were prepared using deionized water as a solvent [13–15]. Water soluble polyoxyethylene oxide (PEO) also added into CMCS solution as an adjuvant to optimization the electrospinning process [16], which is recognized as a non-toxic polymer [17–19].

**Fig. 1:**
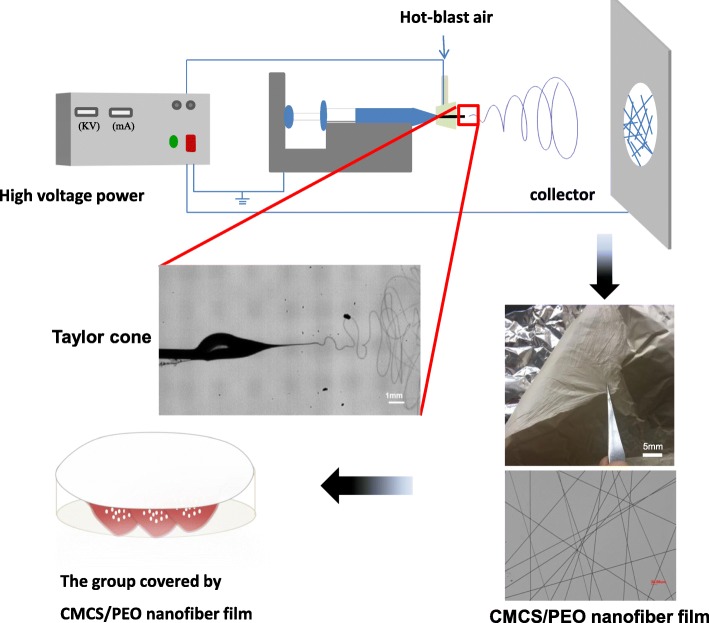
Diagram of the method for preparing the CMCS/PEO nanofiber membrane for strawberries fresh-keeping

Recently, a strategy about fruit fresh-keeping based on chitosan was reported by painting chitosan solution onto the fruit surface to form a wet film, but there existed a few methods to assess fibrous membranes based on electrospinning [20–22]. However, the wet coating film provides contact between the fruit skin and moisture in the air, thus providing an opportunity for bacteria growth and moisture loss. In addition, this coating method requires drying throughout the whole process, which further causes potential damage to the fruit. In this work, we utilize a new type of hand-held electrospinning device for preparation of non-toxic and edible CMCS/PEO nanofiber film (Fig. 1) [5, 23]. The purpose of this research is to evaluate the potential application of chitosan nanofiber films in fruit fresh-keeping and to improve the quality of traditional coatings and extend the shelf life of strawberries.

## Methods/Experimental

### Materials

Organically grown table strawberries were harvested in Laoshan District (Qingdao, China) and took to laboratory as soon as possible; residuals were removed before coating. The strawberries selected are those without mechanical hazard scratches and with similar size, shape, and maturity. CMCS (Mw 80,000 ~ 250,000) with 95% N-deacetylation was purchased from Aoduofuni (Nanjing, China). PEO (Mw ~ 5,000,000) was purchased from Aladdin.

### Preparation of Spinning Solution

Table 1 shows the details of different ratios of the mixed solutions containing CMCS, PEO, and deionized water. Briefly, 3.0 g of CMCS was mixed with 0.16 g, 0.20 g, and 0.25 g PEO, respectively. Then, they were put into 40.0 g deionized water in a bottle of 100 ml. A magnetic stirring was applied for about 4 h at room temperature until the solutions became transparent and homogeneous.

**Table 1:** Solution components for electrospinning

Sample	CMCS/g	PEO/g	Deionized water/g
a	3.0	0.16	40.0
b	3.0	0.20	40.0
c	3.0	0.25	40.0

### Preparation of Nanofiber Membranes

The composite fiber membranes were prepared as follows: 40% relative humidity, syringe needle-to-collector distance of 20 cm, and applied spinning voltage of 20 kV. In this work, a hand-held electrospinning device designed by Qingdao Junada Technology Co. Ltd. was used to prepare CMCS/PEO nanofiber membranes. Figure 1 shows the schematic diagram of the preparation technique and the process of electrospinning.

### Characterization of e-spun Membranes

The morphologies and diameters of nanofibers were characterized by a scanning electron microscopy (SEM; Phenom Pro). The polymer intermolecular structure was determined by a Fourier transform infrared (FTIR) spectrometer (Nicolet iN10; Thermo Fisher Scientific, Waltham). The breathability was measured by a gas transmission rate tester (FX 3300; Zurich).

### Preparation of Preservation Process

**Fig. 2:**
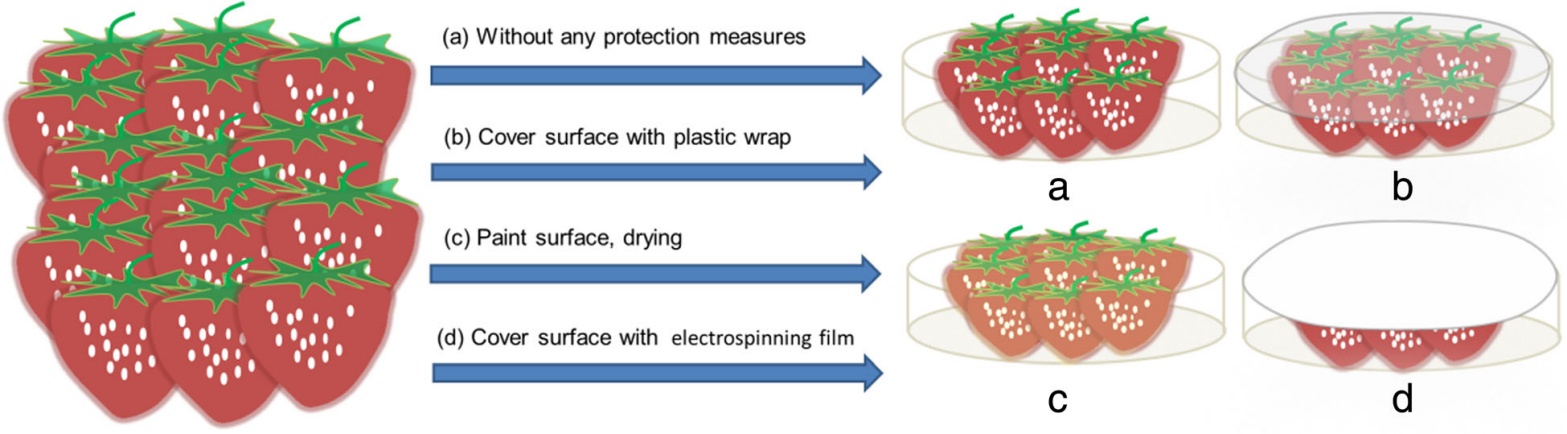
The schematic diagram of strawberry preservation in every group: **a** blank control group in culture dish, **b** group in culture dish covered with plastic film, **c** group with CMCS/PEO solution coatings at surfaces of individual strawberries, and **d** group in culture dish covered with electrospun CMCS/PEO nanofiber film

The strawberries were divided into four groups randomly. Every group had six strawberries in culture dish. The first group was completely exposed to the atmosphere as a blank control group. The second group was wrapped with ordinary household polyethylene plastic film. The third group was painted with the electrospinning solution (PEO: CMCS = 1:20) to form a protective layer with glaze surface outer. In this group, the sample was carefully dried to form a protective film. In addition, electrospun CMCS/PEO nanofiber membrane was used to cover the last group. Finally, these groups were placed at room temperature without sunlight, observed, and recorded at the same time each day. Figure 2 is a schematic diagram of strawberry preservation.

## Results and Discussion

### Morphological Analysis

**Fig. 3:**
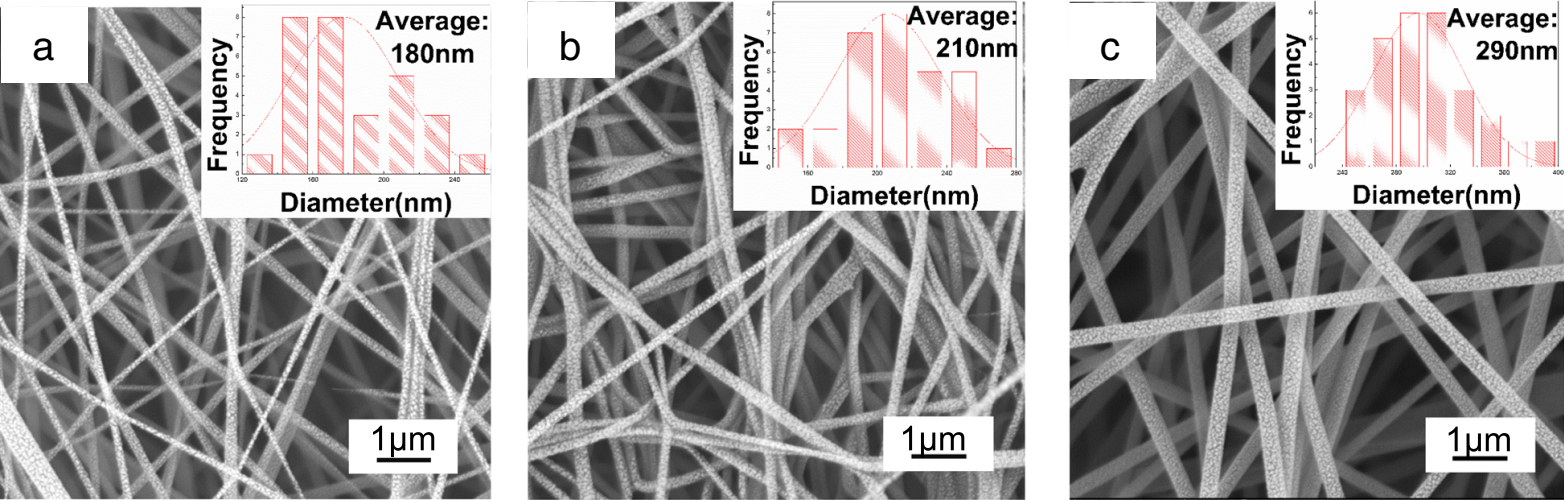
SEM images and fiber diameter distribution of the electrospinning obtained from solutions of **a** PEO: CMCS = 1:24, **b** PEO: CMCS = 1:18, and **c** PEO: CMCS = 1:12

Although pure CMCS solution has high viscosity which can reach up to 400–800 mPa ∙ s, it is still difficult to form fibers by electrostatic fields. The obstacle originates from the molecular structure and solubility of chitin and chitosan, especially for CMCS. For this reason, the fiber forming facilitating polyol binder such as PEO was added into CMCS solution. Under the applied voltage, clear Taylor cone was observed for the CMCS/PEO solutions in the concentration range of 2.5–7.5 wt% (Fig. 1). Figure 3 shows the SEM images and fiber diameter distribution of the composite CMCS/PEO fibers with different ratios. These composite fibers have a cylindrical morphology with fiber diameters about 130–400 nm.

When a smaller amount of PEO was mixed with CMCS, as presented in Fig. 3a (PEO: CMCS = 1:24), the fibers were thinner and inhomogeneous with diameter of 130–280 nm. For solution with PEO: CMCS = 1:18, the average fiber diameter was about 210 nm, and some conglutination between the relatively coarse fibers was observed in Fig. 3b. As the ratio of the PEO increased (PEO: CMCS = 1:12), fairly homogeneous fibers with an average diameter of 290 nm were obtained (Fig. 3c). The nanofiber membrane with ratio of 1:12 of PEO/CMCS was selected as the packaging film, because the solution of 1:12 of PEO/CMCS has a more suitable viscosity for electrospinning and it is easier to form a complete nanofibrous film to cover the fruit, and the electrospun film has a more even breathing intensity due to the uniform size micropores according with the SEM images.

### Infrared Spectroscopy

**Fig. 4:**
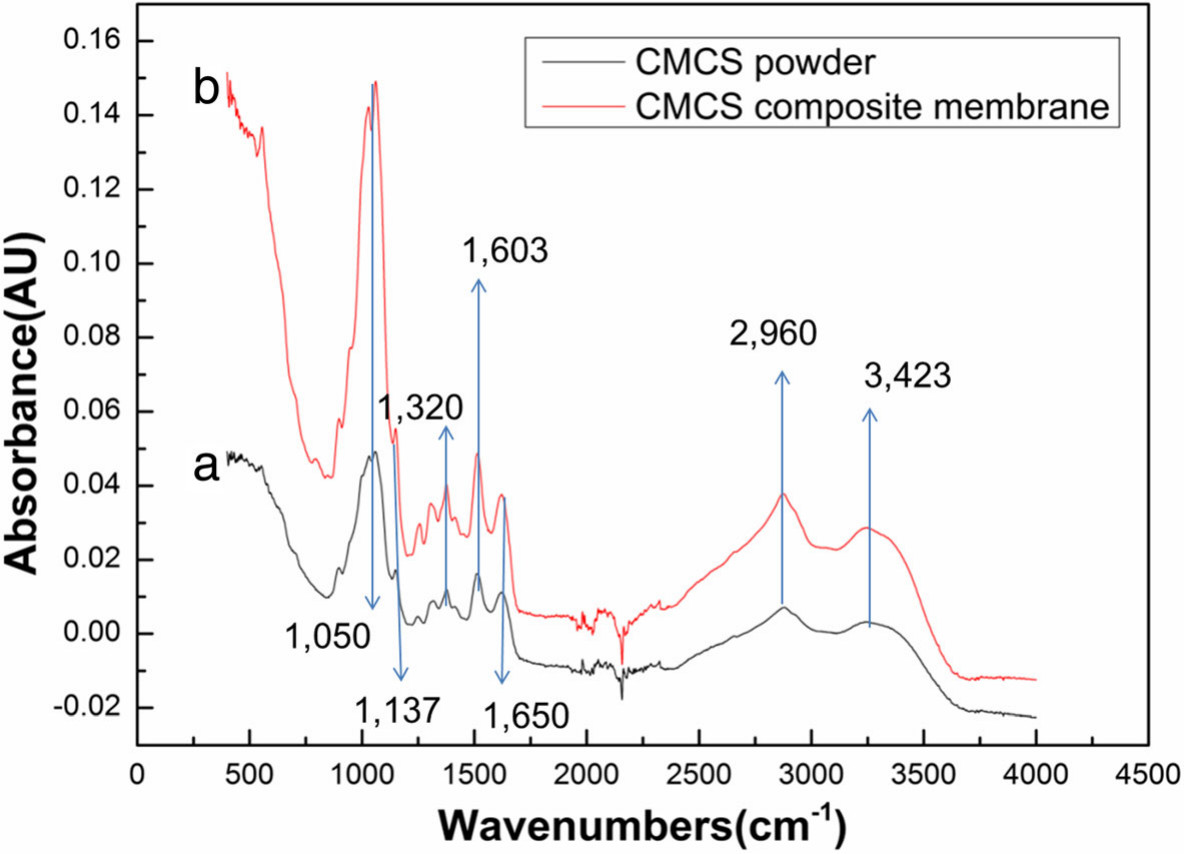
FTIR spectra of the **a** electrospun CMCS powder and **b** electrospun CMCS/PEO composite nanofiber membrane

Figure 4 shows the FTIR spectra of electrospun CMCS powder and CMCS/PEO composite nanofibers. The frequencies and assignments for the pristine CMCS are indicated as follows: the peaks at 1320 cm^−1^, 1137 cm^−1^, and 1050 cm^− 1^ were from C–H bending vibration, glycosidic bond C–O–C, and C–O stretching vibration of CMCS, respectively. In the spectra, new peaks at 1603 cm^−1^ characteristic of carboxylic acid salt (–COO– asymmetrical and symmetrical stretch) appeared, while a shoulder peak around 1650 cm^−1^ indicative of the amino group. Though some difference in the two figures were observed, both of them showed the basic characteristic peaks for CMCS at 3423 cm^−1^ (O–H stretch) and 2960–2970 cm^−1^ (C–H stretch). We can see that the FTIR spectra had no change by an addition of the PEO, which indicated that there was no obvious change of structure between CMCS powder and CMCS/PEO.

### Air Permeability Test

**Fig. 5:**
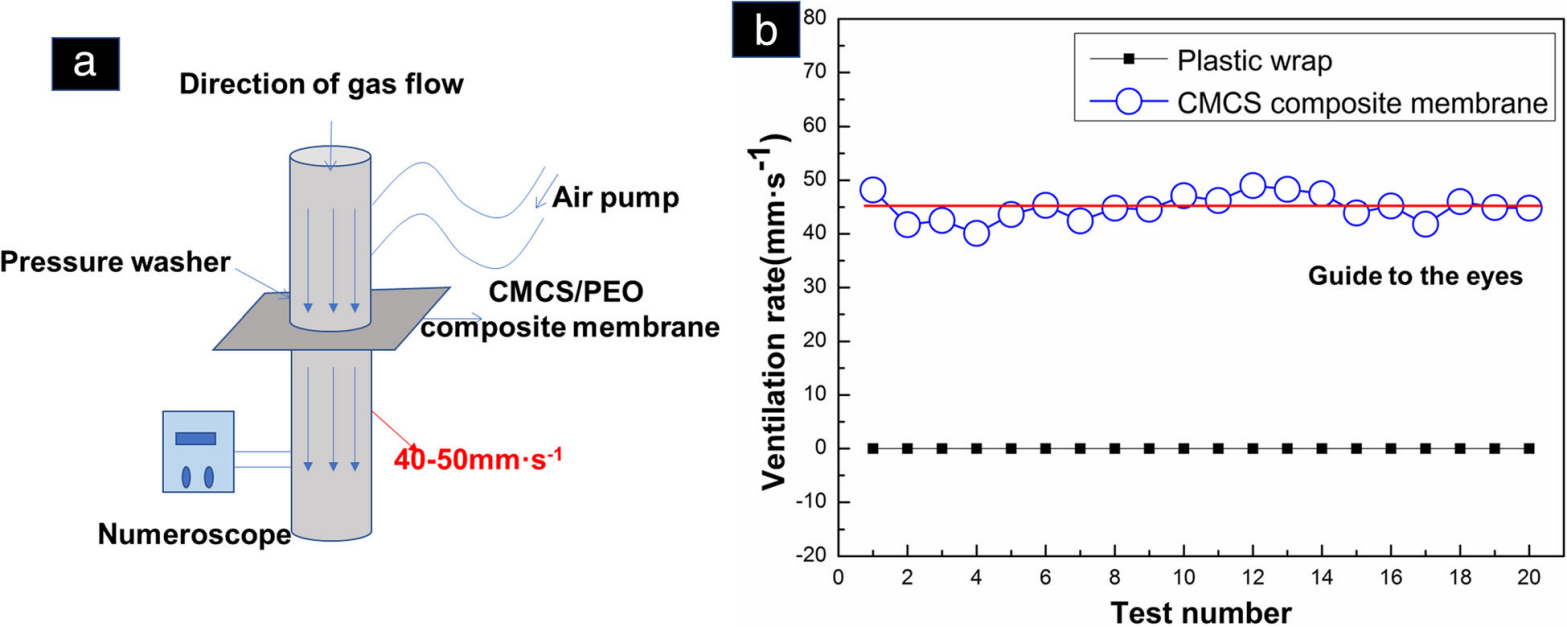
The air permeability of **a** schematic diagram of experimental set-up and **b** the air permeability PEO/CMCS nanofiber membrane with the ratio of 1:12. The data focuses on 45 mm s^−1^. The red line is a guide to the eyes

Many studies have found that permeability is an important factor for preserving fruits. The microporous membrane can promote the exchange of gas inside and outside the package, regulate the concentration of O_2_ and CO_2_, and make the packaged fruits and vegetables have a good storage environment, thus ensuring its quality or being less affected [24]. The certain permeability of the plastic wrap can maintain the appropriate concentration CO_2_ in the confined space. The formation of storage atmosphere can inhibit the respiration of vegetables and extend the shelf life. Provided that breathability is too high, it is easy to make the package oxygen content too high, accelerating breathing of fruits, aging faster, browning, and fading serious [25]. Similarly, poor air permeability or poor air tightness can lead to fruit anaerobic production of alcohol, which ultimately exacerbates fruit rot. [26]. Obviously, the permeability of nanofiber membranes decreases with increasing film thickness. In this experiment, PEO/CMCS composite nanofiber membrane with the ratio of 1: 12 and plastic film were selected for the permeability test. The basic test principle of the device used here is as below (Fig. 5a). Difference in gas pressure at both ends of a circular tube is controlled, 200 Pa in this case. Then measure the air flow rate at the air outlet, so that the greater the air resistance, the lower the air velocity. In the same situation, the measuring result of plastic wrap was 0 mm s^−1^. According to the literature, we know that the air permeability of nylon and other fabrics is between 100 and 300 mm s^−1^ in average [27]. In the measurement of 200 Pa and 20 cm^2^, the measured value of PEO/CMCS composite nanofiber evenly distributed in the 40–50 mm s^−1^ (Fig. 5), indicating that the CMCS/PEO composite membrane had uniform air permeability. In this test, the average film thickness was 0.108 mm. Generally speaking, this breathability is suitable for using as a packaging material with preservation function.

### Antibacterial Test

**Fig. 6:**
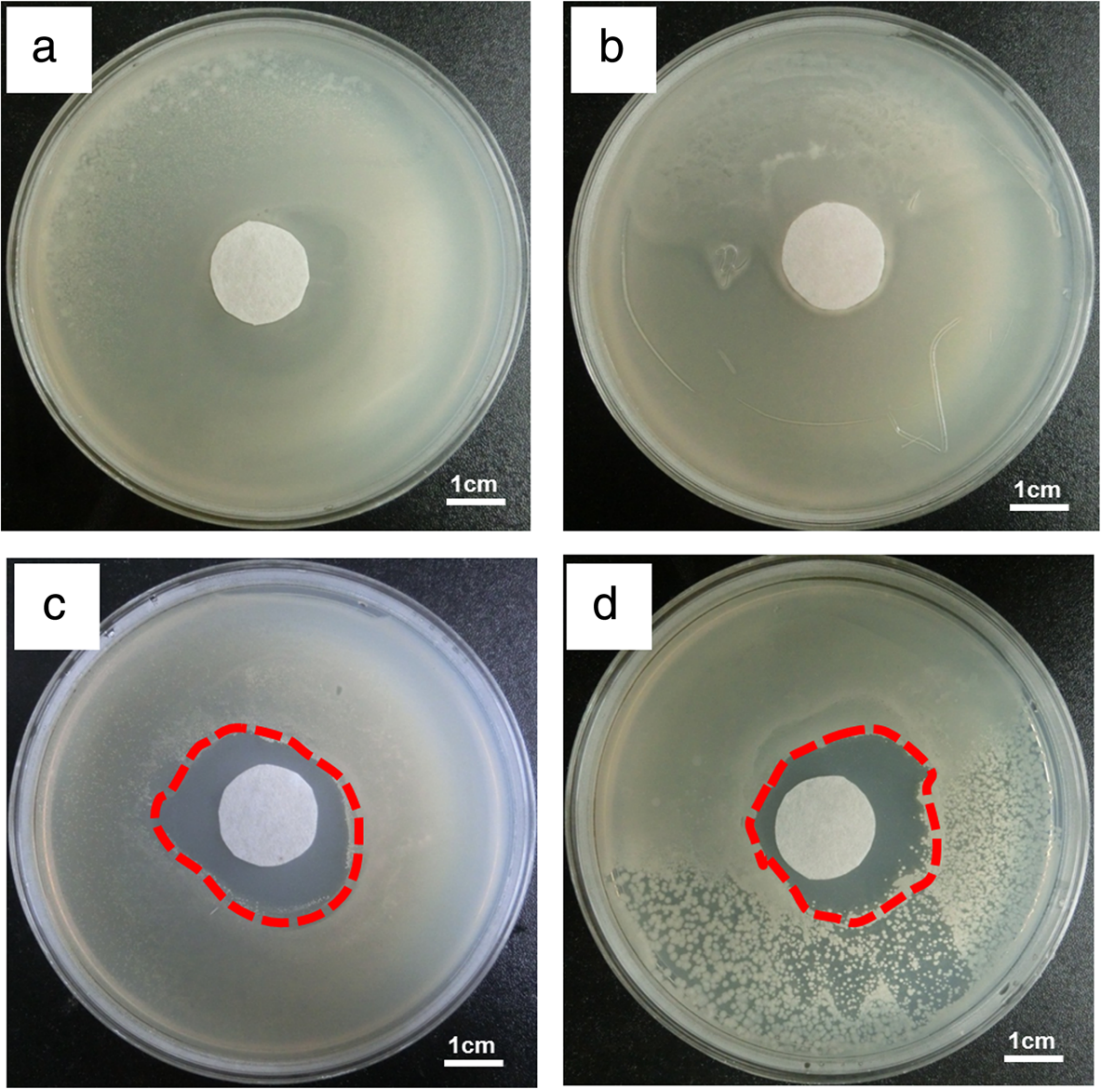
The inhibition of CMCS/PEO nanofibers on Staphylococcus aureus and *Escherichia coli*. **a** *Staphylococcus aureus* with filter paper (control), **b** *Escherichia coli* with filter paper (control), **c** *Staphylococcus aureus* with CMCS/PEO nanofibers, and **d** *Escherichia coli* with CMCS/PEO nanofibers

Presently, many studies have been focused on the antibacterial property of chitosan, but less on the antibacterial property of CMCS. Chitosan has a significant inhibitory effect on many bacteria and fungi, such as *Escherichia coli* and *Staphylococcus aureus*, both of which are the culprits of fruit spoilage [28]. According to investigation, although the antibacterial ability of CMCS is not in direct proportion with its concentration, CMCS showed the strongest ability of antibacterial at the appropriate concentration [29]. It is particularly pointed out that amino of CMCS could inhibit the bacteria after the CMCS was dissolved in the solution by combining the anion [30, 31]. From the viewpoint of bacteriostasis, electrospun CMCS nanofibers are suitable as antimicrobial food packaging material, even if their water solubility limited the application range. As shown in Fig. 6, we performed antibacterial experiments on filter paper and CMCS fiber membranes using *Escherichia coli* and *Staphylococcus aureus*, respectively. The results showed that CMCS/PEO nanofiber membrane had obvious inhibitory effect on these two kinds of bacteria and formed a broad antibacterial ring after 18 h of training. However, the two control groups did not have any bacteriostatic effect in (a) and (b). It is noted that the bacteriostatic rings were not uniform in Fig. 6c, d due to the water solubility and fluidity of CMCS.

### Weight Loss Percentage

The weight-loss ratio could be calculated from the following formula:

Weight loss (%) $$ =\frac{M_0-M}{M_0}\times 100\% $$,

where *M*_0_ is the fresh weight of the strawberries (strawberries are stored for 0 day), and *M* is the weight of samples stored for different days.

**Fig. 7:**
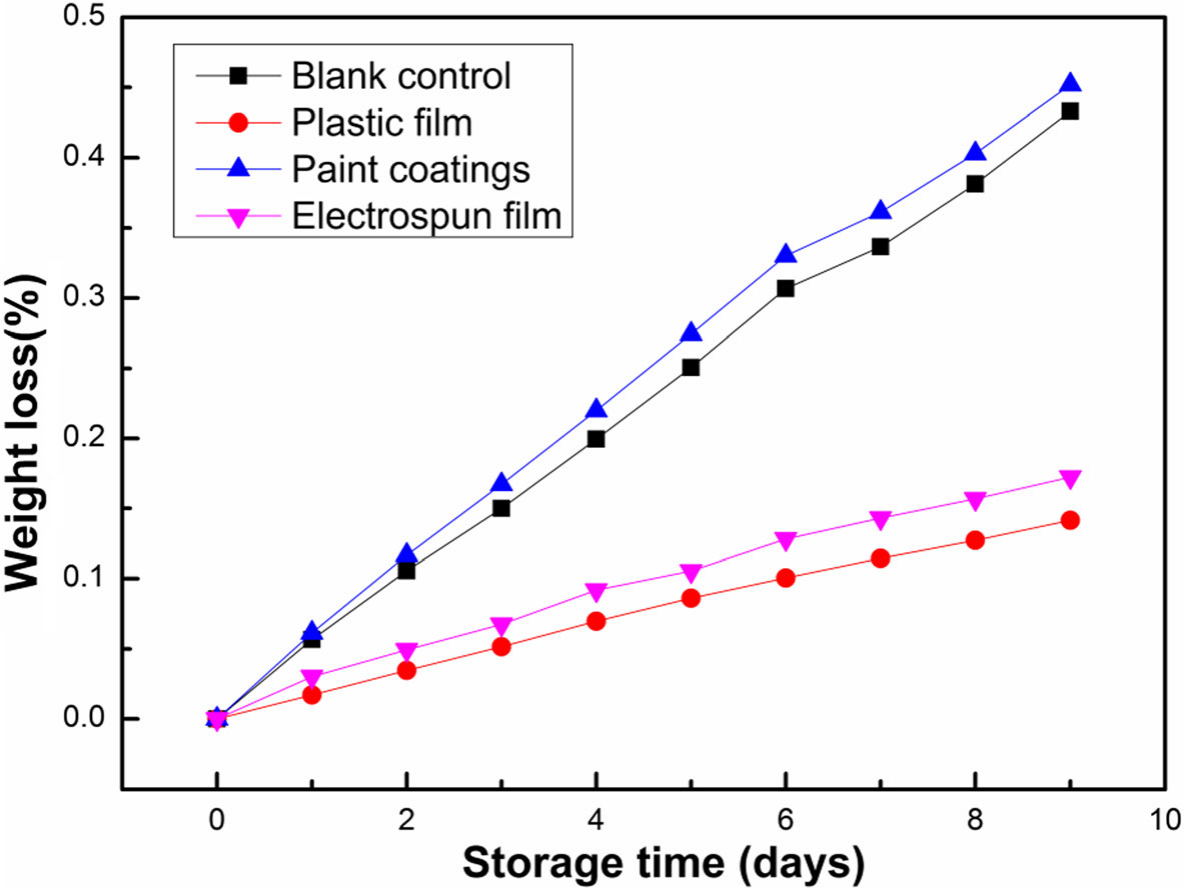
The weight loss ratios of strawberries in different groups during storage at ambient temperature

Weights of different treatment groups were measured at different storage times. As shown in Fig. 7, the blank control group experienced an acceleration of weight loss, which can be attributed to an increase in the fruit’s metabolic activity. Compared with the blank control group, the fruit treated with wrapping by plastic film has quite low weight loss on account of the compactness the plastic film. Apparently, we focused on the group of the CMCS/PEO coating film that the weight loss is more severe. In this case, despite the forming of the CMCS/PEO layer, it leads to physical and direct contact between moisture and fruit surface. With contact of the both, moisture destroyed the outermost natural protection layer of fruits, which in turn resulted in the acceleration in the inside water loss rate. For the group covered by electrospun CMCS/PEO nanofiber film, it showed the fairly good water retention compared with the blank control group and it did not have many effects of the film that the raw materials are hydro soluble.

### Fruit Fresh-Keeping Test

**Fig. 8:**
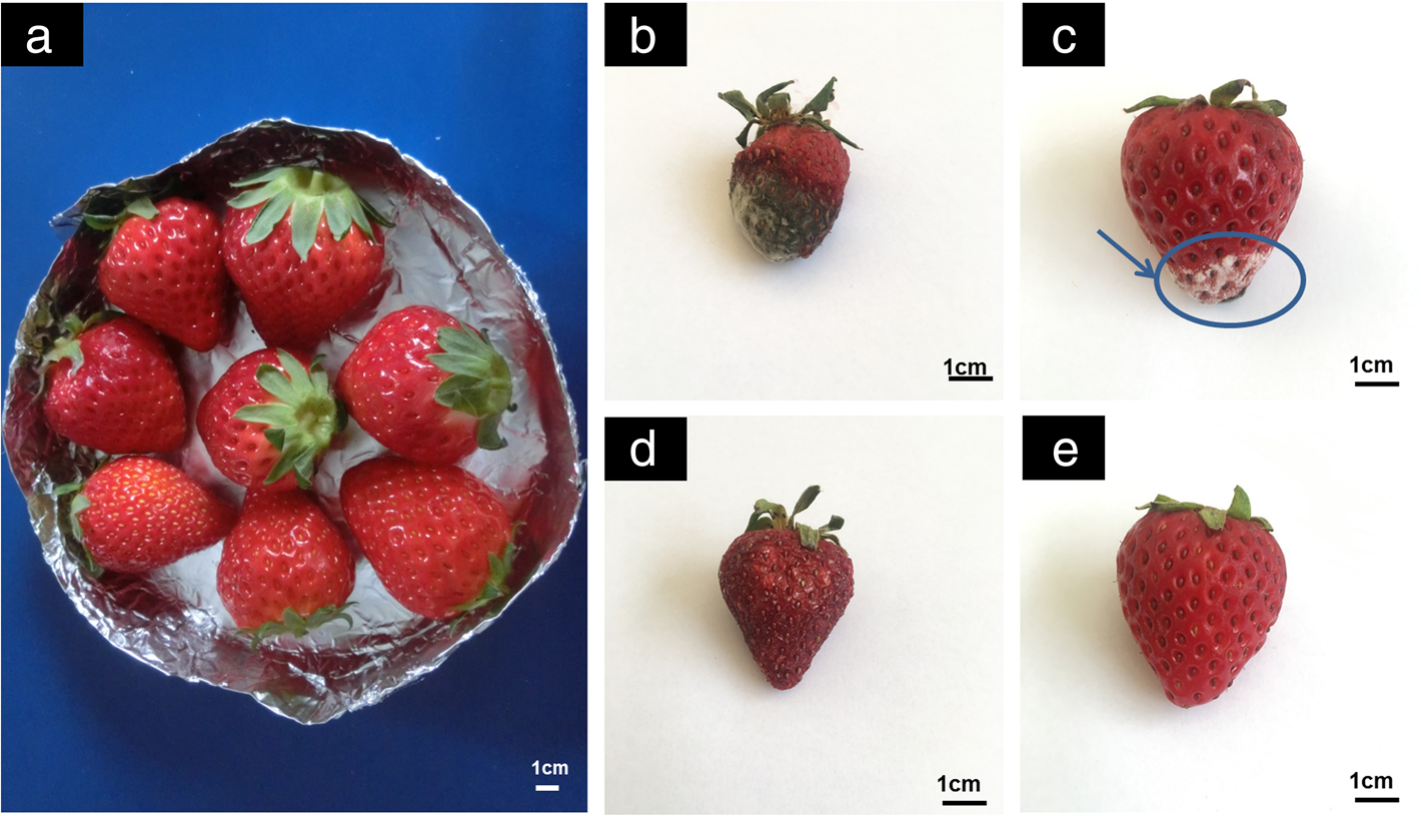
The initial strawberries **a** and the effects of different treatments on appearance of strawberries of the same size after 6 days of storage at ambient temperature: **b** blank control, **c** protected with plastic film, **d** protected by CMCS/PEO paint coatings, and **e** protected with electrospun CMCS/PEO nanofiber film

With regard to fruit fresh-keeping, sensory properties are clearly a significant characteristic as an evaluation criterion. The initial (day 0) sensory properties (color, odor and texture) of these four samples are presented as consistency to the same extent (Fig. 8a). As can be seen from the Fig. 8, throughout storage, colors were dimmed in various degrees in all treatments. The initial full and glossy appearance of the blank control group had largely disappeared, and 70% of the fruit had begun to rot, on account of strawberries that are thinly peeled and rich in juice, being extremely mechanical vulnerable, especially for water loss. The applied example shows that volume had been obviously shrunk to some extent, with quality decrease from 19.59 to 11.10 g for an average of control (Fig. 8b). PE wrapper had some implications in the management of prevention and control of dehydration. In Fig. 8c, strawberries had wilted for a few, with the color became darkened, and mildew appeared on the part of the individual. It is noted that the group of CMCS/PEO paint coatings is mainly darkening and browning (Fig. 8d). Browning is mainly due to the oxidative degradation of ascorbic acid. As mentioned above, the group of those decorated with paint coatings had destroyed skin and the covering layers of the fruits look in bad condition such as the skin was not smooth and severe shrinkage, but without any rot. The results showed that the electrospun CMCS/PEO nanofiber film was effective in preventing diseases and rot and improving the appearance of fruit in the storage in the Fig. 8e. Just like other groups, the strawberries in this group also had a little shrinkage and an aromatic flavor. The causes of off-flavor, in general, can be related to the microbial proliferation and sugar accumulation.

## Conclusions

In summary, we developed a non-poisonous and edible CMCS/PEO nanofiber membrane that not only showed the excellent antimicrobial property but also had admirable air permeability by the hand-held electrospinning device. The CMCS/PEO nanofiber membrane exhibited antibacterial capability to both *Escherichia coli* and *Staphylococcus aureus*. The measured gas permeability was on a scale of 40–50 mm s^−1^ in the 200 Pa. These results indicate that the CMCS/PEO nanofiber membrane may be suitable as packing materials for fruit. Compared with typical conventional coatings, the nanofiber film may have potential applicability. This environmentally friendly technology may provide an alternative approach to the fruit in growing, transporting, and selling.

## Abbreviation

CMCS: Carboxymethyl chitosan
FTIR: Fourier transform infrared
PEO: Polyoxyethylene oxide
SEM: Scanning electron microscopy
